# Living beyond One’s Means

**Published:** 1864-09

**Authors:** H. C. Bowen


### LIVING BEYOND ONE'S MEANS.

BY H. C. BOWEN.

We have once or twice, recently, alluded to a practice prevalent among business-men, *of living beyond their means*, and thus bringing upon themselves a failure, which was no fault of their mode of business, but only of their manner of living. It is not safe to look only at a man's store, to know his standing in business ; you must look also at his house. His splendid profits may entirely merge themselves in his splendid dwelling; so that, if he should suddenly fail, his assets would be found to consist chiefly of carpets, mirrors, frescoes, pictures, marbles, furniture, and a variety of similar articles, all belonging to the inside of a "brown-stone front." Now, if what is poured into the top of a pitcher runs out through a hole in the bottom, it will take continual pouring to keep it full ; a sudden stoppage will leave it empty and dry. We need hardly say, that it takes a large business to support a fine house ; and when the fine house taxes the business to its utmost, a small reverse, which otherwise a man would hardly have felt, may now occasion his ruin. The foundation of a man's fortune is laid on two corner stones—one in his store, the other in his house. If he builds too heavily on either of these, he will have the whole roof down upon him. Many a man who has been known as the "architect of his own fortune," has built unwisely upon one or other of these foundations, and has, at last, been surprised with a worse fall than the tumbling of the State Arsenal !

It is true, the line of difference between living within one's means, and living beyond them, may sometimes be difficult to draw, so as to give the greatest proper limit to free expenditure. For instance, a man may be able to keep a horse and buggy, and live within his means, who, if he were to keep two horses and a carriage, would be living beyond them. A man may keep a fine house in the city, and be well able to afford it, who, as soon as he builds another in the country, is going farther than his money will follow. A man may give an ice-cream party, and not feel it, who, when he gives a fancy-dress ball, will suffer for it a month afterwards. A man may pick his teeth on the steps of the St. Nicholas, and be living frugally within his means, who, if he were once to pay for his dinner at that hotel, would not have a cent left for his supper! When a man is conscious that he is straining a point for a splendid house, or a fast horse, or a grand soiree, or an extravagant table, he may be sure, that *he* is the man who is "living beyond his means!"

The temptations to such extravagance, in such a city as this, are very great. In this respect, the improved architectural taste exhibited in our modern dwelling-houses, has exerted a favorable and also an unfavorable influence on the community—favorable, so far as the progress of art and the culture of the people are concerned; but unfavorable, in having incited a desire for ambitious display, in which men seek to indulge themselves beyond their means. What would have been called an elegant residence, twenty years ago, is now regarded as a mere common three-story house. Compare old, aristocratic Bleecker Street, with new, aristocratic Fifth Avenue! The best houses in Bleecker Street—which were the finest that our fathers or elder brothers ever thought of building—are now mere respectable brick fronts—nothing more! But the least pretentious houses in Fifth Avenue are solid brown stone, or solid white marble—nothing less! This change is, on the whole, an improvement, in the increased beauty of the city; yet it cannot be denied, that the rage for luxurious and ostentatious living, incited and fostered by the introduction of this new element into our private architecture, has never before been so great as now! In fact, nine out of every ten opinions given in accounting for the late commercial crisis, alleged this general extravagance as the cause of the revulsion. When one-third of the merchants of a great city hang one-third of their fortunes upon the lace-mantles of their wives, merely to make a glitter by gas-light at a grand soiree, it is not at all surprising, that the ghost of a mercantile crisis should be seen stalking behind the guests after they leave, in the dead hours of the night! The expense of a well-dressed wife or daughter, in the simple article of jewelry, for a single evening, is oftentimes as much as would originally have bought the entire island of Manhattan, before the time of Peter Stuyvesant! When the "little bills" for these trifles are sent in, and paid, the Crisis may be imagined as bringing up the rear, like Banquo's Ghost!

We write a true epitaph, when we say that many a man's failure has resulted, not from losses in his business, but from losses to which he is blind, because they are hidden in parlor carpets, enameled furniture, and gilded cornices, or in pearl necklaces, topaz brooches, and diamond rings!

### *Living Beyond One's Means.*

We may allude, also, not only to the practice of *living* beyond one's means, but of *doing business* beyond one's means. When, for instance, a new firm, with four or five partners, starting with a capital not exceeding fifty thousand dollars, conduct their business in such a way, that their entire expenses, for the first year, will not be less than thirty thousand, we say that they are doing business beyond their means. If, during the first year, they have large profits to increase their capital, they may come out unharmed. But suppose instead of profits they have losses? Add ten thousand dollars of loss to thirty thousand of expenses, and deducting this from fifty thousand of original investment, what capital remains as the foundation for next year's business? The mere expense of carrying on the *machinery of business*, is the secret of many a man's failure! An ocean steamer cannot be sent across the Atlantic without the continual dropping of oil in her engines; but if the engineer, in lubricating the valves and pistons, instead of using raw linseed, were to lavish upon rod and cylinder the costly ottar of roses, which is said to be worth more than its weight in gold, he might find at the end of his voyage that his oil had cost more than coal and cargo! Now, when a merchant undertakes to oil the wheels of his business with the ottar of roses, he may suddenly find himself at the end of his voyage, before he is over the sea!

We do not mean to say that a man whose fortune is large, should not live freely; or that a man, whose business is large, should not conduct it in splendid and even princely style. Every man should be generous and free-handed to the extent of his means. Otherwise, what possible good can come from a great fortune! For, if a man gains wealth, and uses it only selfishly; if he clothes himself with it as with an golden armor, so that no man's hand may reach to his heart for help; if he heaps up his possessions around him, merely as a wall behind which he is to sit a solitary miser, counting his hoards; he might better never have laid one penny upon the top of another, and have gone through the world a beggar! We advise no meanness of purse, while the purse is full; but we condemn the pretence of fullness, when it is empty! We equally condemn the unthrifty recklessness and extravagance by which men are every day reducing themselves unconsciously from wealth to poverty, and too often, as a natural consequence, from integrity to dishonesty. If, when a man ruins his business, he injured only himself, he would have a perfect right to do it, just as he would have a right to cut off his own fingers, if he pleased! But one man's failure is too often the cutting off of other men's hands! When a great house falls, a hundred others tremble. When a large banking institution in England failed a few years ago, a thousand poor laboring men, widows, and infirm persons, who had invested in it their little all, were ruined! No man has a right to indulge in any extravagance which will injure his success in business, so long as such an injury to himself will be an injury to any one else.

If it be true, that "economy leads to wealth," it must be equally true that extravagance leads to poverty; and almost the greatest extravagance, to which business men are now tempted, is that of ostentatious display. Dr. Franklin said, that "the road to wealth is as plain as the road to market." If Poor Richard were to come again to point it out, he would likely advise that it be not carpeted with Brussels, except at its extreme farther end!

